# Integrative and inclusive genomics to promote the use of underutilised crops

**DOI:** 10.1038/s41467-023-44535-x

**Published:** 2024-01-08

**Authors:** Oluwaseyi Shorinola, Rose Marks, Peter Emmrich, Chris Jones, Damaris Odeny, Mark A. Chapman

**Affiliations:** 1 International Livestock Research Institute, Naivasha Road, Nairobi, Kenya; 2 School of Bioscience, University of Birmingham, Edgbaston, Birmingham, B15 2TT UK; 3 Department of Horticulture, Michigan State University, East Lansing, MI 48824 USA; 4 Plant Resilience Institute, Michigan State University, East Lansing, MI 48824 USA; 5 Norwich Institute for Sustainable Development, School of Global Development, University of East Anglia, England, NR4 7TJ UK; 6 Center of Excellence in Genomics and Systems Biology, ICRISAT, Patancheru, 502324 Telangana India; 7 Biological Sciences, University of Southampton, Life Sciences Building 85, Highfield Campus, Southampton, SO17 1BJ UK

**Keywords:** Plant breeding, Agricultural genetics, Genetic variation, Research management

## Abstract

Underutilised crops or orphan crops are important for diversifying our food systems towards food and nutrition security. Here, the authors discuss how the development of underutilised crop genomic resource should align with their breeding and capacity building strategies, and leverage advances made in major crops.

## Teaser

Underutilised crops are important for diversifying food systems. The genomics of these crops should not be done in isolation but should align with their breeding and capacity building strategies while leveraging advances made in major crops.

Before the advent of agriculture, humans regularly consumed nearly 7000 plant species, yet only ~250 species have been fully domesticated, and only ~30 of these provide ~95% of global calorie intake^1^. Investment in this narrow set of crops has reduced nutritional and ecological resilience. There are a variety of lesser known domesticated or semi-domesticated crops that have significant potential for making our food systems more diverse and resilient, and whilst being locally important, barriers to their wider adoption exist. We use the term “underutilised” to describe these crops, rather than other terms which imply they are abandoned (“neglected” or “orphan”), unimportant (“minor”), or simply from a certain place (“indigenous”). Each of these terms would encompass a different group of crops, and by using the term “underutilised”, we wish to focus on the unfulfilled potential of these crops to contribute to global nutrition.

**Fig. 1:**
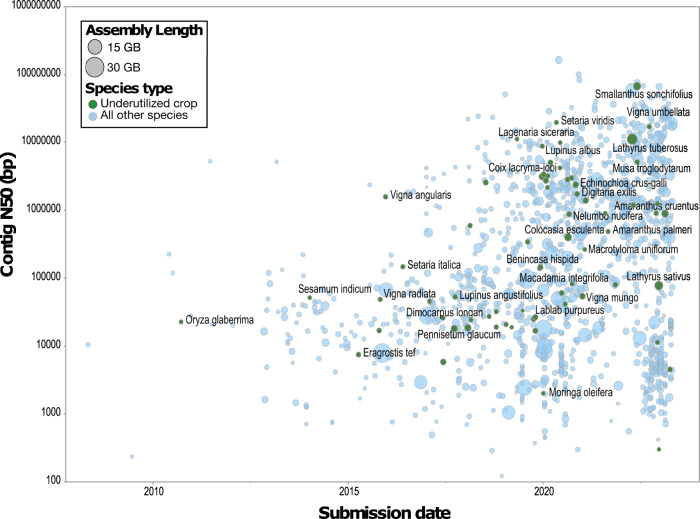
Summary of underutilised crops genomics studies. Assembly metrics by submission date for 1324 land plant species with publicly available genome assemblies from 2008 to 2023. An example (non-exhaustive) set of underutilised crops are highlighted in green while other plant species are highlighted in blue. Genome size is represented by bubble size. Source data are provided as a Source Data file, which consists of Supplementary Table 1 from Marks et al.^15^ updated with data for assemblies published till July 2023.

Although initially lagging, the genomics of underutilised crops has gathered pace due to technological advances in genome sequencing, assembly, and annotation (Fig. 1). The availability of genomic resources is proving useful in elucidating underutilised crop histories - their polyploidization^2^, domestication^3^ and artificial selection histories^4^, among others. Genomics has also shed light on the genetic architecture of important agronomic and end-use traits like tuber quality in greater yam^5^, and the unique climate resilience of crops like tepary bean that is adapted to desert habitats^6^. Recent reviews provide detailed description of progress made in underutilised crop genomics^7,8^. There have also been several reviews on the broad use, policy and ethical considerations around underutilised crops in general^9–11^.

**Fig. 2:**
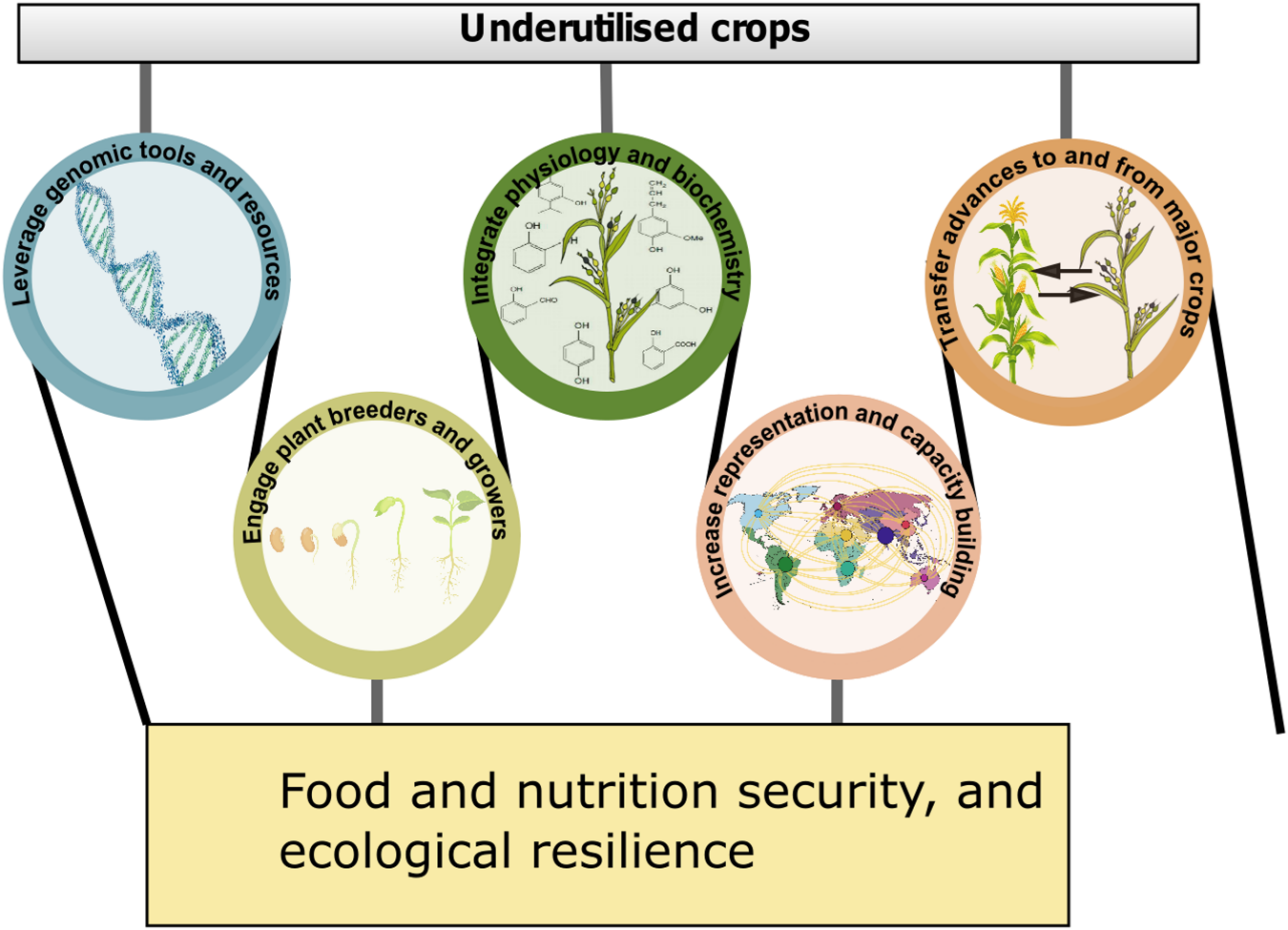
Integrated and inclusive genomics for underutilised crops. To achieve a diversified and resilient food system through the utilisation of underutilised crops, the genomics of these crops must align with their breeding programmes and end-users’ needs, promote participation and capacity building of the communities that depend on them, and leverage information from other fields and crops. The map present in this Figure was adapted from Marks et al.^15^. The other images in the Figure were purchased from Shutterstock.

In this article, we focus our discussion on how to maximise positive impact in this new era of underutilised crop genomics. As genome sequencing platforms become more and more accessible, there is a tendency to “just sequence everything”. However, we argue that the development of underutilised crop genomic resources should not be done in isolation. Instead, such efforts should be aligned with the overall breeding strategies of the target crops as well as the needs and perceptions of the communities that depend on these crops, while leveraging existing advances in major crops (Fig. 2). We describe these points in detail below and provide examples where applicable.

## Underutilised crops genomics efforts should align with their seed system and breeding strategies

While improved seeds of most major crops are easily accessible through formal seed systems (involving structured public and/or private sector breeding and distribution channels), underutilised crops are predominantly distributed in informal seed systems between smallholder farmers, complicating rapid adoption of improved materials. Although genomics may not be immediately relevant across the entire seed systems, initial engagement of stakeholders particularly at the germplasm assessment and breeding stages can significantly increase the attractiveness, profitability, and uptake of underutilised crops. This is especially because underutilised crops’ farmers and end-user perceptions are sometimes based on tacit traditional knowledge that are not always captured by metric traits used in formal breeding. Incorporating this tacit knowledge in genomic screening as demonstrated by Woldeyohannes et al.^12^ and Gesesse et al.^13^ can facilitate a deeper understanding of the adaptive potential and end-use value of existing genetic diversity in farmers’ fields and conserved diversity in gene banks. This understanding can be used to integrate formal and farmer-participatory models to identify, prioritise or develop adapted and farmer-preferred germplasm. This model does not necessarily require formal plant breeding, as valuable germplasm (landraces) identified from genomic-assisted participatory screening can be made widely and readily available to farmers through integrated seed systems as suggested in the “Seeds for Needs” model^14^.

To maximise impact and relevance of underutilised crop genomics, the choice of germplasm used in generating genomic resources should align with the demand from the breeding and end-use communities. This is particularly important in the selection of cultivars for reference genome assemblies, which should be representative of important founder parents, have rich genetic resources (markers, genetic maps) or harbour demand-led traits that are locally important. For instance, in sequencing the genome of greater yam, Bredeson et al.^5^ sequenced a breeding line that is frequently used as a parent in the greater yam breeding program and that is moderately resistant to anthracnose, an important disease in this crop.

## Representation and capacity building will promote the use of underutilised crop genomes

As is the trend in plant genomics more broadly^15^, genomic research on underutilised crops is dominated by affluent nations and institutions. Some exceptions include the whole genome sequencing of International Crops Research Institute for the Semi-Arid Tropics (ICRISAT) mandate crops **— **coordinated largely from India^16^, the greater yam genome from Nigeria^5^, the Lima bean genome from Colombia^17^, and the recent lablab genome from East Africa^3^. Given the overwhelming significance of underutilised crops to many indigenous communities^18^, genomics projects on underutilised crops should be used to develop research capacities in these regions. The ability to utilise a genome is just as important as having access to the genome. While it is not essential for everyone to become proficient with genomic data, systems with overlapping expertise and effective communication chains across interest groups are needed to fully realise the potential of genomic data and tools in underutilised crops. While short-term (days) capacity building efforts are useful, we argue that longer-term, practical and in-country trainings are more impactful, especially when customised to fill specific gaps within the local context. These efforts should not be afterthoughts but should be considered at the inception of genomics projects on underutilised crops. Researchers from communities with a good understanding of farmer and consumer preferences should co-design and co-lead genomic research on the target crops.

A growing number of international collaborations and consortia (e.g Africa BioGenome Project^19^, African Orphan Crops Consortium^20^, and CABANA/Capacity Building for Bioinformatics in Latin America) are now promoting inclusion and capacity building in genomics research on under-represented crops. This inclusivity and diversity help to promote curiosity-driven research and the integration of local knowledge enriches research questions leading to more relevant, trusted and translatable research outputs, better adoption of recommendations, and increased ownership and stewardship of the resulting resources.

## Advances in major crops should be used to fast-track underutilised crops genomics

There is a need to accelerate the rate of genetic improvement in underutilised crops. We have a broad knowledge of the genes and molecular pathways underlying many agriculturally relevant traits in major crops, and a suite of tools (transgenics, gene editing, genomic selection, speed breeding) have been developed to improve such crops. These tools should be leveraged to transfer knowledge and fast-track the de novo domestication and improvement of underutilised crops.

Genomic selection and speed breeding have emerged as some of the most prominent breeding innovations that significantly reduce breeding time and cost. These tools could be especially valuable for helping small breeding programmes (as is typical of underutilised crops) accelerate genetic gains using limited resources^21,22^. Importantly, genomic selection and speed breeding protocols that have been established for many major crops could be easily transferred to closely related underutilised crops to achieve immediate impact. Furthermore, genomic advances in major crops have highlighted the importance of coalescing multiple de novo assemblies into a crop pangenome to present a wider picture of the gene content of a species. Core genome regions are found in all accessions, but may make up only half of the pangenome^7^, while dispensable regions could contain adaptive variation, for example in foxtail millet^23^. If applied to underutilised crops, these approaches would give a much more complete picture of genomic variation.

Gene editing provides an unprecedented opportunity to accelerate the improvement and domestication of underutilised crops. Knowledge of gene function from related major crops could inform the selection of candidate gene targets in underutilised crops, while taking into account that gene function is not always conserved across species. The simplicity, versatility and cost-effectiveness of the CRISPR/Cas systems has democratised gene editing, and is already showing potential for domesticating underutilised crops, as was demonstrated for groundcherry^24^ and African rice^25^ by targeting genes that were firstly studied in major crops.

Despite this potential, the use of gene editing particularly in underutilised crops should be carefully considered in light of the traditional and cultural value of these crops to indigenous communities. The lack of efficient and genotype-independent transformation and regeneration protocols stalls the broad adoption of gene editing in many underutilised crops^26^. Also, licensing issues still present a major hurdle to the use of the CRISPR/Cas systems in varietal development^27^. As part of the capacity building effort suggested above, we advocate for dedicated initiatives and funding to promote the transfer of technical knowledge (in transformation and regeneration) and establishment of well-resourced, and appropriately licensed tissue culture labs for the use of transformation-enhancing systems (including morphogenic genes like *WUSCHEL*, *BABY BOOM* and *GRF-GIF*) and gene editing in communities working on underutilised crops.

## Linking genomics with physiology and biochemistry to unlock the resilience and nutraceutical potential of underutilised crops

Many underutilised crops are adapted to degraded, marginal or otherwise poor soils, and tolerate drought, cold or heat stresses. They are often the only crops standing, when most staple crops have failed, giving them the reputation of famine or insurance crops. Many indigenous communities use underutilised crops to promote healthy living or treat ailments due to their rich nutrient and nutraceutical composition^28^. Underutilised crops are therefore a reservoir of novel adaptive genetic, physiological and biochemical variation.

There is a need to integrate underutilised crop genomics with plant physiology and biochemistry to build a more cohesive picture of their adaptation and metabolic phenotypes at the cell, organ, plant and species levels. As genomics is becoming more accessible, detailed physiological and metabolic studies on underutilised crops are needed to achieve this integration. Studies like those of Edwards et al.^29^ and Moghaddam et al.^6^ demonstrate the value of combining genomics with physiology and biochemistry to uncover adaptation and metabolic phenotypes in underutilised crops. Several adaptive genes or alleles from underutilised crops have already been shown to provide climate resilience in model plants or staple crops^28^.

### Conclusion

Underutilised crop genomics has come of age but should not stand alone. Translating genomic advances into viable pathways towards food and nutritional security will require integrating different systems (basic research, breeding, and seed systems), participants (e.g., academics, breeders and indigenous communities), tools (e.g., gene editing, genomic selection, speed breeding) and disciplines (e.g., genomics, physiology, biochemistry). Research into both underutilised and major crops would benefit each other: underutilised crops could inform the improvement of resilience and nutritional traits in major crops while domestication, yield and agronomy improvement in underutilised crops would benefit from knowledge acquired in major crops. It is also important to note that sharing of germplasm and digital sequence information (DSI) is a part of this process, and a review of international policies on germplasm access and benefit sharing is needed to promote research into underutilised crops^11,30^.

### Supplementary information

Supplementary Information
